# Insight into the Modification of Phosphatidylcholine with n-3 Polyunsaturated Fatty Acids-Rich Ethyl Esters by Immobilized MAS1 Lipase

**DOI:** 10.3390/molecules24193528

**Published:** 2019-09-29

**Authors:** Xiumei Wang, Xiaoli Qin, Xiuting Li, Zexin Zhao, Bo Yang, Yonghua Wang

**Affiliations:** 1 Key Laboratory of Loquat Germplasm Innovation and Utilization, Fujian Province University, College of Environmental and Biological Engineering, Putian University, Putian 351100, China; wxm890626@163.com; 2 School of Bioscience and Bioengineering, South China University of Technology, Guangzhou 510006, China; bizexin-zhao@mail.scut.edu.cn (Z.Z.); yangbo@scut.edu.cn (B.Y.); 3 College of Food Science, Southwest University, Chongqing 400715, China; qinxiaoli66@163.com; 4 Beijing Advanced Innovation Center for Food Nutrition and Human Health, Beijing Technology and Business University, Beijing 100048, China; lixt@btbu.edu.cn; 5 Guangdong Research Center of Lipid Science and Applied Engineering Technology, School of Food Science and Engineering, South China University of Technology, Guangzhou 510640, China

**Keywords:** immobilized MAS1 lipase, phosphatidylcholine, n-3 polyunsaturated fatty acids, ethyl esters, transesterification

## Abstract

This study reported the modification of phosphatidylcholine (PC) with n-3 polyunsaturated fatty acids (PUFA)-rich ethyl esters (EE) by immobilized MAS1 lipase-catalyzed transesterification in the solvent-free system. Effects of n-3 PUFA-rich EE/PC mass ratio, enzyme loading, reaction temperature, and water dosage on the incorporation of n-3 PUFA into PC were investigated, respectively. The results indicate that the maximum incorporation of n-3 PUFA into PC reached 33.5% (24 h) under the following conditions: n-3 PUFA-rich EE/PC mass ratio of 6:1, enzyme loading of 20%, reaction temperature of 55 °C, and water dosage of 1.0%. After 72 h of reaction, the incorporation of n-3 PUFA into PC was 43.55% and the composition of the reaction mixture was analyzed by ^31^P nuclear magnetic resonance (NMR). The results show that the reaction product consisted of 32.68% PC, 28.76% 1-diacyl-*sn*-glycero-3-lysophosphatidylcholine (*sn*-1 LPC), 4.90% 2-diacyl-*sn*-glycero-3-lysophosphatidylcholine (*sn*-2 LPC), and 33.60% *sn*-glycero-3-phosphatidylcholine (GPC). This study offers insight into the phospholipase activity of immobilized MAS1 lipase and suggests the extended applications of immobilized MAS1 lipase in the modification of phospholipids for industrial purpose.

## 1. Introduction

Long-chain n-3 polyunsaturated fatty acids (PUFA), especially eicosapentaenoic acid (EPA) and docosahexaenoic acid (DHA), are proven to have beneficial effects on human health such as decreasing the risk of cardiovascular disease, preventing cancer, and inhibiting inflammation [1,2,3,4]. Studies have shown that n-3 PUFA-rich phospholipids (PL) have higher bioavailability and oxidation stability than n-3 PUFA-rich triacylglycerols and n-3 PUFA-rich ethyl esters (EE) [5,6]. In addition to its nutritional value, n-3 PUFA-rich PC is also known to possess good biological activities such as anti-atherosclerosis, anti-inflammatory, ameliorating lipid accumulation, improving learning capabilities, and promoting osteogenesis [7,8,9,10,11,12,13]. Thus, n-3 PUFA-rich PC is an attractive product and has been widely used as emulsifiers, nutritive substrates, a pharmacologically active agent in an emulsion, a component in pharmaceutical compositions, and a component in lipid particles in the foods, cosmetics, agriculture, and pharmaceutical industries [14,15,16,17,18]. Therefore, there is great interest in the synthesis of n-3 PUFA-rich PC.

Synthesis of n-3 PUFA-rich PC could be carried out using chemical or enzymatic approaches. Compared with chemical methods, enzymatic production of n-3 PUFA-rich PC is effective due to its beneficial advantages such as high catalytic efficiency, mild reaction conditions, and high positional selectivity [19]. Enzymatic acidolysis of PC with n-3 PUFA and enzymatic transesterification of PC with n-3 PUFA-rich EE have been widely employed to produce n-3 PUFA-rich PC [20,21,22]. Nevertheless, n-3 PUFA are easy to oxidize during the reactions and derived from their EE form [23,24], resulting in a complicated process. Besides, n-3 PUFA-rich EE are the main PUFA product in the market. Therefore, enzymatic transesterification of PC with n-3 PUFA-rich EE is used to catalyze the synthesis of n-3 PUFA-rich PC [25,26].

Enzymatic transesterification reactions can be performed by lipases or phospholipases. Although phospholipase A_1_ exhibited higher catalytic efficiency than lipases in the transesterification reactions [27], the cost of phospholipase is expensive and its source is limited. Besides, few studies on the production of n-3 PUFA-rich PC by lipase-catalyzed transesterification of PC with n-3 PUFA-rich EE have been reported. Moreover, it was found that the incorporation of n-3 PUFA into PC was still less than 30% when lipases were used to catalyze the transesterification reactions in the solvent-free system [28]. Therefore, it is necessary to explore other lipases with better catalytic ability for the synthesis of n-3 PUFA-rich PC through transesterification of n-3 PUFA-rich EE with PC.

**Scheme 1:**
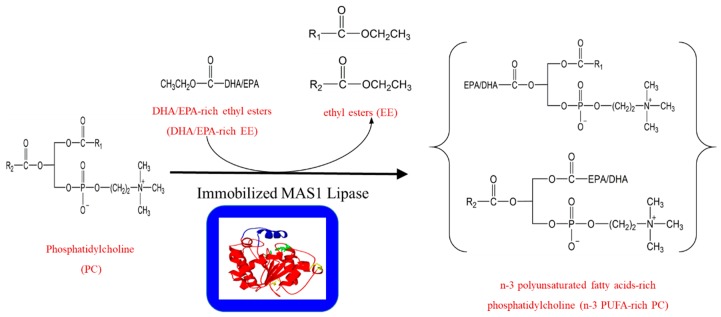
Immobilized MAS1 lipase-catalyzed transesterification of n-3 PUFA-rich EE with PC for the production of n-3 PUFA-rich PC.

In recent decades, immobilized lipases have attracted considerable attention by virtue of their good durability and recyclability, high stability, activity and selectivity, good resistant to environmental changes when compared with free lipases [29,30]. Therefore, an immobilized MAS1 lipase using XAD1180 resin as a carrier, which was from marine *Streptomyces* sp. strain W007 [31], was used to catalyze transesterification of n-3 PUFA-rich EE with PC for the production of n-3 PUFA-rich PC in the solvent-free system in this study (Scheme 1). The effects of n-3 PUFA-rich EE/PC mass ratio, enzyme loading, reaction temperature, and water dosage on the incorporation of n-3 PUFA into PC were investigated, respectively. Finally, ^31^P nuclear magnetic resonance (NMR) was employed to analyze the composition of the final reaction product.

## 2. Results and Discussion

### 2.1. Effect of n-3 PUFA-Rich EE/PC Mass Ratio

**Figure 1:**
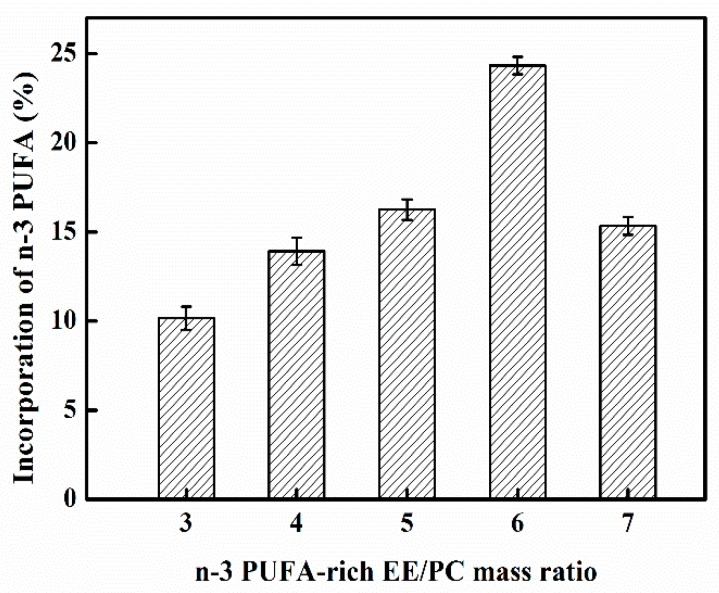
Effects of n-3 PUFA-rich EE/PC mass ratio on the incorporation of n-3 PUFA into PC. Reaction conditions were as follows: enzyme loading of 15% (*w*/*w*), water dosage of 1% (*w*/*w*), reaction temperature of 55 °C, reaction time of 24 h under N_2_.

The effects of n-3 PUFA-rich EE/PC mass ratio on the incorporation of n-3 PUFA into PC were investigated in the range from 3:1 to 7:1. The results are given in Figure 1. When n-3 PUFA-rich EE/PC mass ratio varied from 3:1 to 6:1, the incorporation of n-3 PUFA into PC increased. After that, the incorporation of n-3 PUFA into PC decreased when n-3 PUFA-rich EE/PC mass ratio was further increased from 6:1 to 7:1. The maximum incorporation of n-3 PUFA into PC reached 24.33% at the n-3 PUFA-rich EE/PC mass ratio of 6:1. This probably indicates that excess n-3 PUFA-rich EE not only can decrease mass transfer, but also can increase the solubility of PC and the concentration of n-3 PUFA. Moreover, too high substrate mass ratio could result in difficulties of separation of the products and increase the cost of the process. Therefore, n-3 PUFA-rich EE/PC mass ratio was fixed at 6:1 in the subsequent experiments.

### 2.2. Effect of Enzyme Loading

**Figure 2:**
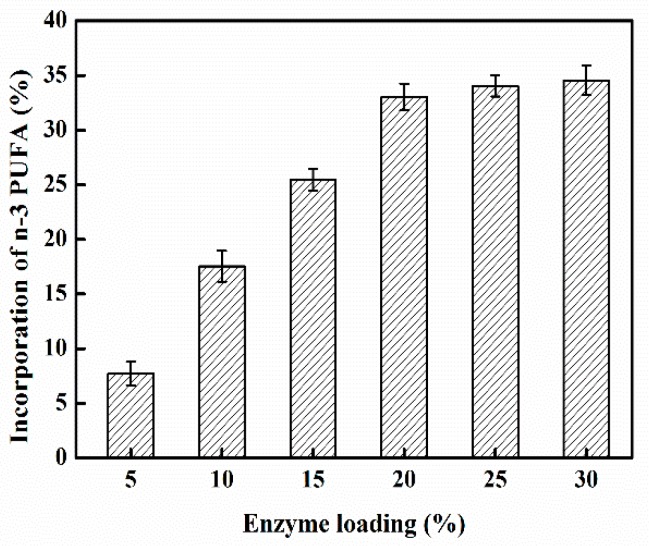
Effects of enzyme loading on the incorporation of n-3 PUFA into PC. Reaction conditions were as follows: n-3 PUFA-rich EE/PC mass ratio of 6:1, water dosage of 1% (*w*/*w*), reaction temperature of 55 °C, and reaction time of 24 h under N_2_.

The effects of enzyme loading on the incorporation of n-3 PUFA into PC are shown in Figure 2. There was an obvious increase of the incorporation of n-3 PUFA into PC from 7.73% to 33.03%, when enzyme loading was increased from 5% to 20%. The incorporation of n-3 PUFA into PC reached 33.03% at enzyme loading of 20%. When enzyme loading exceeded 20%, the incorporation of n-3 PUFA into PC increased slightly. Therefore, taking into account the cost of the reaction, enzyme loading of 20% was selected for further study.

### 2.3. Effect of Reaction Temperature

**Figure 3:**
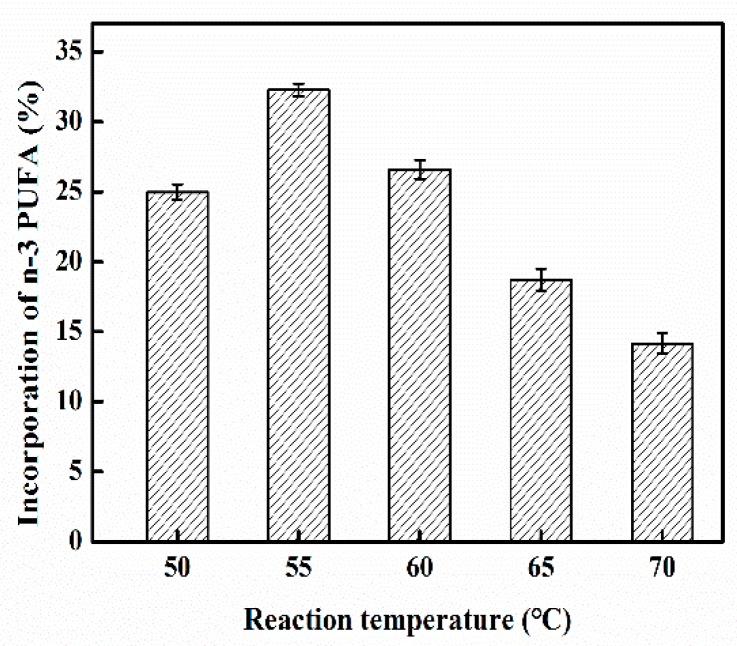
Effects of reaction temperature on the incorporation of n-3 PUFA into PC. Reaction conditions were as follows: n-3 PUFA-rich EE/PC mass ratio of 6:1, enzyme loading of 20% (*w*/*w*), water dosage of 1% (*w*/*w*), and reaction time of 24 h under N_2_.

The effects of reaction temperature on the incorporation of n-3 PUFA into PC were evaluated and the results are presented in Figure 3. The incorporation of n-3 PUFA into PC increased when reaction temperature increased from 50 to 55 °C. The highest incorporation of n-3 PUFA (32.26%) was achieved at 55 °C. However, the incorporation of n-3 PUFA into PC decreased when reaction temperature was further increased from 55 to 70 °C. This probably because of the deactivation of the immobilized MAS1 lipase when the reaction temperature exceeded 55 °C. Therefore, reaction temperature of 55 °C was used for the following experiments.

### 2.4. Effect of Water Dosage

**Figure 4:**
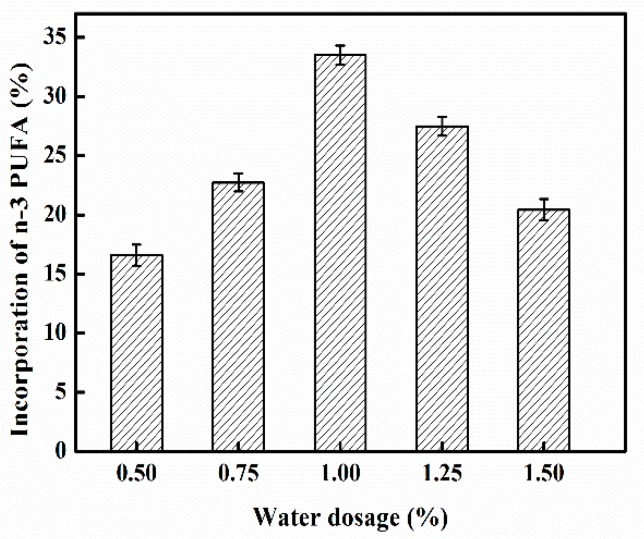
Effects of water dosage on the incorporation of n-3 PUFA. Reaction conditions were as follows: n-3 PUFA-rich EE/PC mass ratio of 6:1, enzyme loading of 20% (*w*/*w*), reaction temperature of 55 °C, and reaction time of 24 h under N_2_.

The effects of water dosage on the incorporation of n-3 PUFA into PC were investigated and the results are shown in Figure 4. The incorporation of n-3 PUFA into PC increased with increasing water dosage from 0.5% to 1.0%. The maximum incorporation of n-3 PUFA (33.49%) was observed at water dosage of 1.0%. Then, the incorporation of n-3 PUFA into PC decreased when water dosage was further increased from 1.0% to 1.5%. This is probably because of the undesired hydrolysis reactions when excess water was added into the reaction mixtures. Therefore, the optimal water dosage for the modification of PC with n-3 PUFA-rich EE was 1.0%.

### 2.5. Time Course of Transesterification of PC with n-3 PUFA-Rich EE by Immobilized MAS1 Lipase

**Figure 5:**
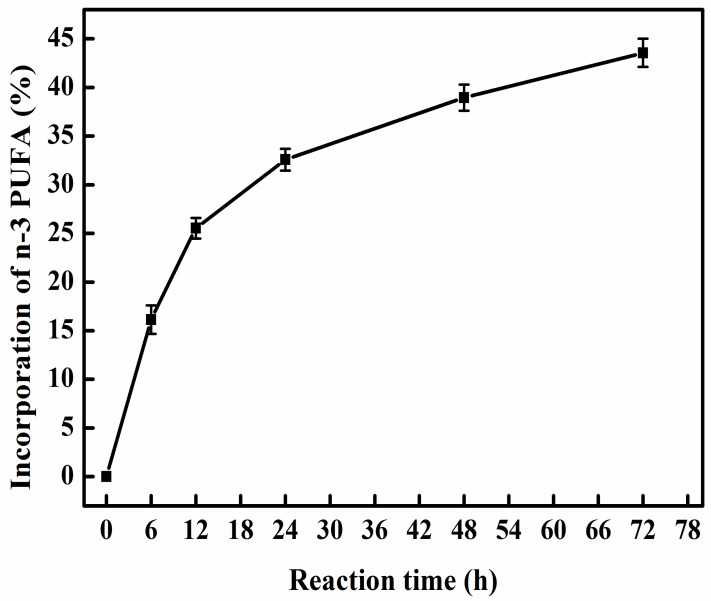
The time course of immobilized MAS1 lipase-catalyzed transesterification of PC with n-3 PUFA-rich EE. Reaction conditions were as follows: n-3 PUFA-rich EE/PC mass ratio of 6:1, enzyme loading of 20% (*w*/*w*), reaction temperature of 55 °C, and water dosage of 1.0% under N_2_.

The time course of immobilized MAS1 lipase-catalyzed transesterification of PC with n-3 PUFA-rich EE was performed under the optimal conditions and the results are given in Figure 5. As the reaction proceeded, the incorporation of n-3 PUFA into PC increased with time. The incorporation of n-3 PUFA into PC increased rapidly in the first 24 h. The incorporation of n-3 PUFA into PC was 33.5% at 24 h. However, the incorporation of n-3 PUFA increased relatively slowly but steadily in the next 48 h. The incorporation of n-3 PUFA into PC reached 43.55% at 72 h.

The composition of the substrate and the final reaction product after 72 h of reaction was analyzed by ^31^P NMR and the results are given in Table 1. The substrate consisted of 97.96% PC, 1.76% *sn*-1 LPC and 0.28% *sn*-2 LPC. After 72 h of reaction, PC content decreased to be 32.68%, whereas *sn*-1 LPC, *sn*-2 LPC and GPC content increased to be 28.76%, 4.90% and 33.60%, respectively. 

The n-3 PUFA incorporation (DHA + EPA + DPA) into PC obtained by immobilized MAS1 lipase was 33.5% at 24 h and 43.55% at 72 h under the optimized conditions in the solvent-free system in this study. It has been reported that the n-3 PUFA incorporation (DHA + EPA + DPA) into PC was 32.6% at 24 h when immobilized PLA_1_ was used to catalyze the transesterification reactions in the solvent-free system [27]. Thus, it could be concluded that the catalytic efficiency of immobilized MAS1 lipase was similar to that of immobilized PLA_1_ when the transesterification reactions were performed for 24 h. However, lower n-3 PUFA incorporation (<35%) was obtained at 72 h when Lipozyme RM IM was used to catalyze transesterification of soybean lecithin and ethyl esters of polyunsaturated fatty acids in the absence of organic solvent and additives [28]. Besides, the n-3 PUFA incorporation (DHA + EPA + DPA) into PC and PC yield obtained by immobilized PLA_1_ in another study were 32.31% and 32.1% at 72 h in the solvent-free system, respectively [22]. On the other hand, although the incorporation of lauric acid (LA) into PC reached 44.2% during the modification of PC using immobilized Rhizopus oryzae in n-hexane [25], the fatty acids used in the study were different from those in this study. In addition, it was reported that the maximum PC yield under the optimized conditions was 14.3% when phospholipase A_2_ was used to catalyze transesterification of PC with eicosapentaenoic acid ethyl ester in toluene [26]. Thus, it could be concluded that, although enzymatic modification of PC in these two studies was performed in organic solvents, the results obtained by immobilized MAS1 lipase in the solvent-free system in this study are still better than those obtained by other lipase and phospholipase in organic solvents in these two studies. Therefore, these results indicate that immobilized MAS1 lipase exhibited relatively high catalytic activity and is a promising biocatalyst for the modification of phospholipids.

## 3. Materials and Methods

### 3.1. Materials

Lipase MAS1 was produced according to the method previously described [32]. DHA/EPA-rich ethyl esters (EE) were kindly donated by Sinomega Biotech Engineering Co., Ltd. (Zhejiang, China). Granulated soy phosphatidylcholine (PC, purity > 95%, used as reaction substrate) was purchased from Avanti Polar-Lipids, Inc. (Alabasta, AL, USA). Standards of 37-component fatty acid methyl esters (FAME) mix (C_4_-C_24_), phosphatidylcholine (PC), lysophosphatidylcholine (LPC) and *sn*-glycero-3-phosphatidylcholine (GPC) were sourced from Sigma-Aldrich. Triphenylphosphate and deuterated chloroform were purchased from Aladin Reagent (Shanghai, China). n-Hexane of chromatographic grade were obtained from Kermel Chemical Reagent Co., Ltd. (Tianjin, China). All other chemicals were of analytical grade unless otherwise stated. In this study, the composition of n-3 PUFA was the sum of the composition of EPA, DPA and DHA.

### 3.2. Immobilization of Lipase MAS1

A non-commercial immobilized MAS1 lipase was prepared using XAD1180 resin (hydrophobic support) as a carrier in our laboratory, as described in our previous report [33]. Firstly, the supernatant of crude lipase MAS1 (75 mg/g resin) and an equal volume of sodium phosphate buffer (0.02 mol/L, pH 8.0) were mixed and added to a conical flask containing XAD1180 resin. Then, the flask was placed in a shaking water-bath with a speed of 200 rpm at 30 °C for 8 h. After that, the resulting immobilized MAS1 lipase was recovered by filtration and washed several times with sodium phosphate buffer (0.02 mol/L, pH 8.0) until no protein was detected in the filtrate. Finally, the obtained immobilized MAS1 lipase was dried under vacuum at 40 °C for 8 h and stored in sealed vials at 4 °C until use. 

The esterification activity of immobilized MAS1 lipase was determined according to the method previously described [34]. Briefly, immobilized MAS1 lipase was incubated with 20 mM 1-propanol, 20 mM lauric acid and 3% water (*w/w*, with respect to total reactants) at 60 °C with a speed shaking of 200 r/min for 10 min. Then, samples (30 μL) were withdrawn and mixed with n-heptane (970 μL). The propyl ester was analyzed using gas chromatography (GC) equipped with a column OV351 (60 m × 0.32 mm × 0.10 μm) according to the method of Wang et al. [35]. Basing on the above method, the esterification activity of immobilized MAS1 lipase was detected to be 2000 U/g.

### 3.3. Transesterification of PC with EPA/DHA-Rich EE

PC (1 g) and different amount of DHA/EPA-rich EE (1:3, 1:4, 1:5, 1:6, and 1:7 of PC/EE mass ratio) were mixed in 25 mL conical flask. Then, water dosage (0.5%, 0.75%, 1.0%, 1.25%, and 1.5% *w*/*w*, with respect to total reaction substrates) and immobilized MAS1 lipase (5%, 10%, 15%, 20%, 25%, and 30% *w*/*w*, with respect to total reaction substrates) were added into the reaction substrates, respectively. After that, the flasks were incubated at various temperatures (50, 55, 60, 65, and 70 °C) with a speed of 200 rpm under N_2_. Samples were withdrawn periodically for GC and ^31^P NMR analysis.

### 3.4. Analysis of FA Composition by GC

Before the analysis, separation of PC and LPC from the reaction mixtures was carried out on a thin layer chromatography plate using a mixture of chloroform, methanol, acetic acid, and water (75:40:8:3, v/v/v/v) as the developing solvent as described previously [36]. Then, the substrate (DHA/EPA-rich EE), the scraped PC and LPC bands were separately methylated to FAME according to previous report [37]. Finally, the analyses of the FA composition of the substrate, PC and LPC in the reaction mixtures was carried out using an Agilent 7890A GC equipped with a capillary column CP-Sil 88 (60 m× 0.25 mm × 0.2 μm) according to the literature [38]. In our study, the incorporation of n-3 PUFA (%) was calculated as follows:

(1) $$\mathrm{Incorporation}\;\mathrm{of}\;n-3\;\mathrm{PUFA}\;\left(\%\right)=\frac{n-3\;\mathrm{PUFA}\;\mathrm{content}\;\mathrm{in}\;\mathrm{PL}}{\mathrm{total}\;\mathrm{FA}\;\mathrm{content}\;\mathrm{in}\;\mathrm{PL}}\times 100\%$$

### 3.5. Analysis of PL Composition in the Final Products by ^31^P NMR

Phospholipids precipitation was obtained from the reaction mixtures as previously described [39]. Then, ^31^P NMR was used to analyze the PL composition in the reaction mixtures. Triphenylphosphate was used as the internal standard. The detailed analysis was carried out according to the method described previously [27]. MestReNova software (Mestrelab Research SL, Santiago de Compostela, Spain) was utilized to analyze the data. The area percentages of GPC, *sn*-1 LPC, *sn*-2 LPC, and PC were obtained from the integration of signal response of the NMR spectra. The relative *sn*-1 LPC and *sn*-2 LPC content were defined as the content of *sn*-1 LPC and *sn*-2 LPC in the total LPC, respectively.

### 3.6. Statistical Analysis

Each experiment was repeated three times. Then, an ANOVA procedure was used to determine significant differences among the measured values. The data were expressed as the means ± standard deviations (SD).

## 4. Conclusions

PC was successfully modified by incorporation of n-3 PUFA derived from EE using immobilized MAS1 lipase in the solvent-free system. The maximum n-3 PUFA incorporation into PC was 33.5% at 24 h under the optimized conditions. After 72 h of reaction, the PC content decreased to 32.68% and its n-3 PUFA incorporation was 43.55%. These results indicate that immobilized MAS1 lipase is a promising biocatalyst with relatively high catalytic activity for the modification of phospholipids. It is well known that the hydrolysis reaction is an unavoidable side reaction during transesterification of phospholipids. Moreover, the reaction mechanism of immobilized MAS1 lipase-catalyzed transesterification of PC with n-3 PUFA-rich EE is still unknown. Therefore, further research will focus on studying the reaction mechanism and how to inhibit the hydrolysis reaction.

## Figures and Tables

**Table 1:** The composition of phospholipids of the substrate PC and structured PC after 72 h of reaction.

Phospholipids	Content (%)	
	Original PC	Structured PC
PC	97.96 ± 0.45	32.68 ± 0.32
*sn*-1 LPC	1.76 ± 0.23	28.76 ± 0.58
*sn*-2 LPC	0.28 ± 0.22	4.90 ± 0.21
GPC	-	33.66 ± 0.67

Original PC refers to the substrate (granulated soy phosphatidylcholine with C_16_ to C_18_ fatty acids). Structured PC refers to original PC and n-3 PUFA-rich PC. LPC, lysophosphatidylcholine; GPC, *sn*-glycero-3-phosphatidylcholine.

## Abbreviations

PC	phosphatidylcholine
LPC	lysophosphatidylcholine
n-3 PUFA	n-3 polyunsaturated fatty acids
*sn*-1 LPC	1-diacyl-*sn*-glycero-3-lysophosphatidylcholine
GPC	*sn*-glycero-3-phosphatidylcholine
*sn*-2 LPC	2-diacyl-*sn*-glycero-3-lysophosphatidylcholine
NMR	nuclear magnetic resonance
EPA	eicosapentaenoic acid
DHA	docosahexaenoic acid
PL	phospholipids
EE	ethyl esters
FAME	fatty acid methyl esters
GC	gas chromatography
